# Comparison of immune microenvironments between primary tumors and brain metastases in patients with breast cancer

**DOI:** 10.18632/oncotarget.22110

**Published:** 2017-10-27

**Authors:** Rin Ogiya, Naoki Niikura, Nobue Kumaki, Hiroyuki Yasojima, Tsutomu Iwasa, Chizuko Kanbayashi, Risa Oshitanai, Michiko Tsuneizumi, Ken-ichi Watanabe, Akira Matsui, Tomomi Fujisawa, Shigehira Saji, Norikazu Masuda, Yutaka Tokuda, Hiroji Iwata

**Affiliations:** ^1^ Department of Breast and Endocrine Surgery, Tokai University School of Medicine, Kanagawa, Japan; ^2^ Department of Pathology, Tokai University School of Medicine, Kanagawa, Japan; ^3^ Department of Surgery, Breast Oncology, National Hospital Organization Osaka National Hospital, Osaka, Japan; ^4^ Department of Medical Oncology, Kindai University School of Medicine, Osaka, Japan; ^5^ Department of Breast Oncology, Niigata Cancer Center Hospital, Niigata, Japan; ^6^ Department of Breast Surgery, Shizuoka General Hospital, Shizuoka, Japan; ^7^ Department of Breast Surgery, Hokkaido Cancer Center, Sapporo, Japan; ^8^ Department of Surgery, National Hospital Organization, Tokyo Medical Center, Tokyo, Japan; ^9^ Department of Breast Oncology, Gunma Prefectural Cancer Center, Gunma, Japan; ^10^ Department of Medical Oncology, Fukushima Medical University, Fukushima, Japan; ^11^ Department of Breast Oncology, Aichi Cancer Center Hospital, Nagoya, Japan

**Keywords:** breast cancer, brain metastases, tumor infiltrating lymphocytes, PD-L1, immune microenvironment

## Abstract

**Background:**

Immune checkpoint inhibitors are reported to be effective in patients with brain metastases. However, detailed characteristics of the brain metastasis immune microenvironment remain unexplored.

**Results:**

The median tumor-infiltrating lymphocyte (TIL) category in brain metastases was 5% (1–70%). In 46 pair-matched samples, the percentages of TILs were significantly higher in primary breast tumors than in brain metastases (paired t-test, *P* < 0.01). The numbers of CD4/CD8/Foxp3-positive cells were significantly higher in primary breast tumors than in brain metastases (paired *t*-test, *P* < 0.05 for all antibodies). In patients with triple-negative breast cancer specifically, low TIL numbers were associated with significantly shorter overall survival compared to high TIL numbers (log-rank test, *P* = 0.04).

**Materials and Methods:**

We retrospectively identified 107 patients with breast cancer and brain metastases who had undergone surgery between 2001 and 2012 at 8 institutions, and collected 191 samples including brain metastases alone and primary tumors with pair-matched brain metastasis samples. Hematoxylin and eosin-stained slides were evaluated for TILs and categorized according to the extent of staining. Immunohistochemistry for CD4, CD8, Foxp3, PD-L1, PD-L2, and HLA class I was also performed.

**Conclusions:**

There are significantly fewer TILs in brain metastases than in primary breast tumors.

## INTRODUCTION

Breast cancer is the second most common cause of brain metastases [1]. Brain metastases occur later during the course of metastatic disease and have a profoundly negative effect on survival despite extensive treatment. The median survival after a diagnosis of brain metastasis in patients with breast cancer is approximately 15 months (range: 1–55 months) [2, 3]. Thus, new therapeutic options are urgently needed to improve the prognoses of patients with brain metastases.

In the past, the brain was considered an immune-privileged organ; however, many studies show that this immune privilege is not absolute but is relative to that of other organs [4]. Disruption of the blood-brain barrier (BBB) and a change in the composition of the extracellular matrix by central nervous system tumors can render the BBB leaky at the tumor site [5]. The intact brain contains almost no lymphocytes; however, T and B cells have been observed in the milieus of brain metastases [6]. Therefore, the unique features of brain metastases compared to those of extracranial lesions must be considered prior to treatment with immune-modulating therapy.

The presence of tumor-infiltrating lymphocytes (TILs) has recently been associated with favorable long-term outcomes in breast cancer [7]. Previous studies have reported that a higher TIL count at baseline is associated with a greater likelihood of a complete pathological response after neoadjuvant chemotherapy (NAC), particularly in human epidermal growth factor receptor-2 (HER2)-positive and triple-negative (TN; estrogen receptor [ER]/progesterone receptor [PR]/HER2-negative) breast cancers [8]. In addition, the presence of TILs in residual disease after NAC is associated with better prognosis in TN breast cancers [9]. In a previous clinical trial, pharmacological inhibition of the immune checkpoint protein, programmed death 1 (PD-1) was reported to be effective against melanoma, lung cancer, and certain types of breast cancer [10]. Furthermore, programmed death-ligand 1 (PD-L1) expression in tumor cells was found to be predictive of the response to PD-1 or PD-L1 inhibitors in the treatment of lung cancer, although some methodological problems (i.e., discordance between staining conditions, antibodies, fixation conditions, timing of biopsies, and cut-off values for positive vs. negative staining) were noted [11]. PD-1 inhibitors also showed activity against brain metastases in patients with melanoma and lung cancer [12]. However, whether PD-L1 expression in metastatic tumors can predict responses to PD-1 or PD-L1 inhibitors in patients with brain metastases has yet to be determined.

The immune microenvironments of brain metastases arising from breast tumors have been investigated [13, 14] but have not been thoroughly compared to those of the primary breast tumors. In this study, we compared the TIL counts and immune system-related characteristics such as levels of CD4, CD8, Foxp3, PD-L1, PD-L2, and HLA class I antigen between primary breast tumors and the corresponding brain metastases via immunohistochemistry to better characterize the immune microenvironment of brain metastases.

## RESULTS

The clinicopathological characteristics of the 107 patients with breast cancer and brain metastases are detailed in Table 1. The median follow-up time was 13 months (range: 1–152 months) after the initial brain metastasis diagnosis; 20 patients (19%) were alive at the last follow-up visit.

**Table 1:** Patient characteristics

	Total (*N* = 107)	
Age at breast cancer diagnosis mean (range)	51	(22–73)
ER status		
Positive	44	(41%)
Negative	55	(51%)
Unknown	8	(7%)
PR status		
Positive	30	(28%)
Negative	69	(64%)
Unknown	8	(7%)
HER2 status		
Positive	45	(42%)
Negative	54	(50%)
Unknown	8	(7%)
Subtype		
Luminal HER2-negative	27	(25%)
Luminal HER2-positive	15	(14%)
HER2-enriched	29	(27%)
Triple-negative	26	(24%)
Unknown	10	(9%)
Histological grade		
G1	4	(4%)
G2	23	(21%)
G3	34	(32%)
Unknown	46	(43%)
Chemotherapy before brain metastases surgery		
Metastatic	48	(45%)
Neoadjuvant^*^	15	(14%)
Adjuvant^*^	70	(65%)
No	12	(11%)
Unknown	10	(9%)
Number of brain metastases		
3 or less	81	(76%)
More than 3	18	(17%)
Unknown	8	(7%)
Treatment for first brain metastases		
Surgery	91	(85%)
STI	10	(9%)
WBI	6	(6%)

^*^Neoadjuvant/Adjuvant is referring to the treatment timing of the primary breast tumor.ER, estrogen receptor; PR, progesterone receptor; HER2, human epidermal growth factor receptor-2; Luminal, ER-positive and PR-positive type; STI, stereotactic irradiation, WBI, whole-brain irradiation.

**Figure 1:**
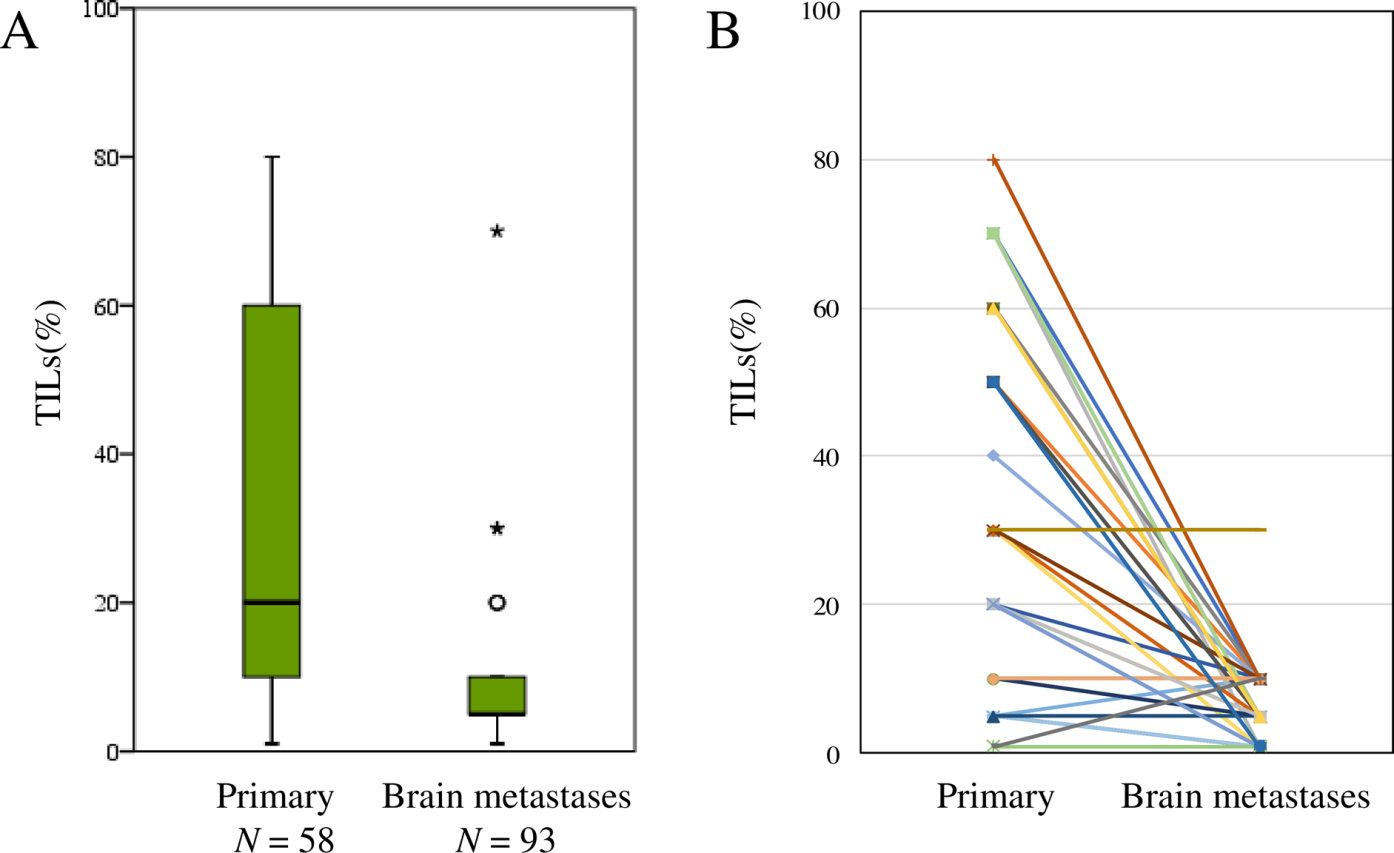
Distribution of tumor-infiltrating lymphocytes (TILs) in primary tumors and brain metastases **(A)**, and a comparison of TILs between primary tumors and brain metastases in 46 pair-matched cases **(B)**.

Fourteen brain metastasis specimens were excluded from our analysis because of low quality or small quantity. The median TIL categories (based on the extent of staining) for primary tumors (*N* = 58) and brain metastases (*N* = 93) were 20% and 5% (ranges: 1–80% and 1–70%), respectively (Figure 1A). Based on the effect of radiotherapy, the median TIL categories for brain metastases for which the first therapy was radiotherapy (*N* = 14) and surgery (*N* = 79) were both 5% (ranges: 1–30% and 1–70%, respectively). There was no significant difference in TILs between the radiotherapy vs. surgery (as first therapy) groups (*P* = 0.72).

**Figure 2:**
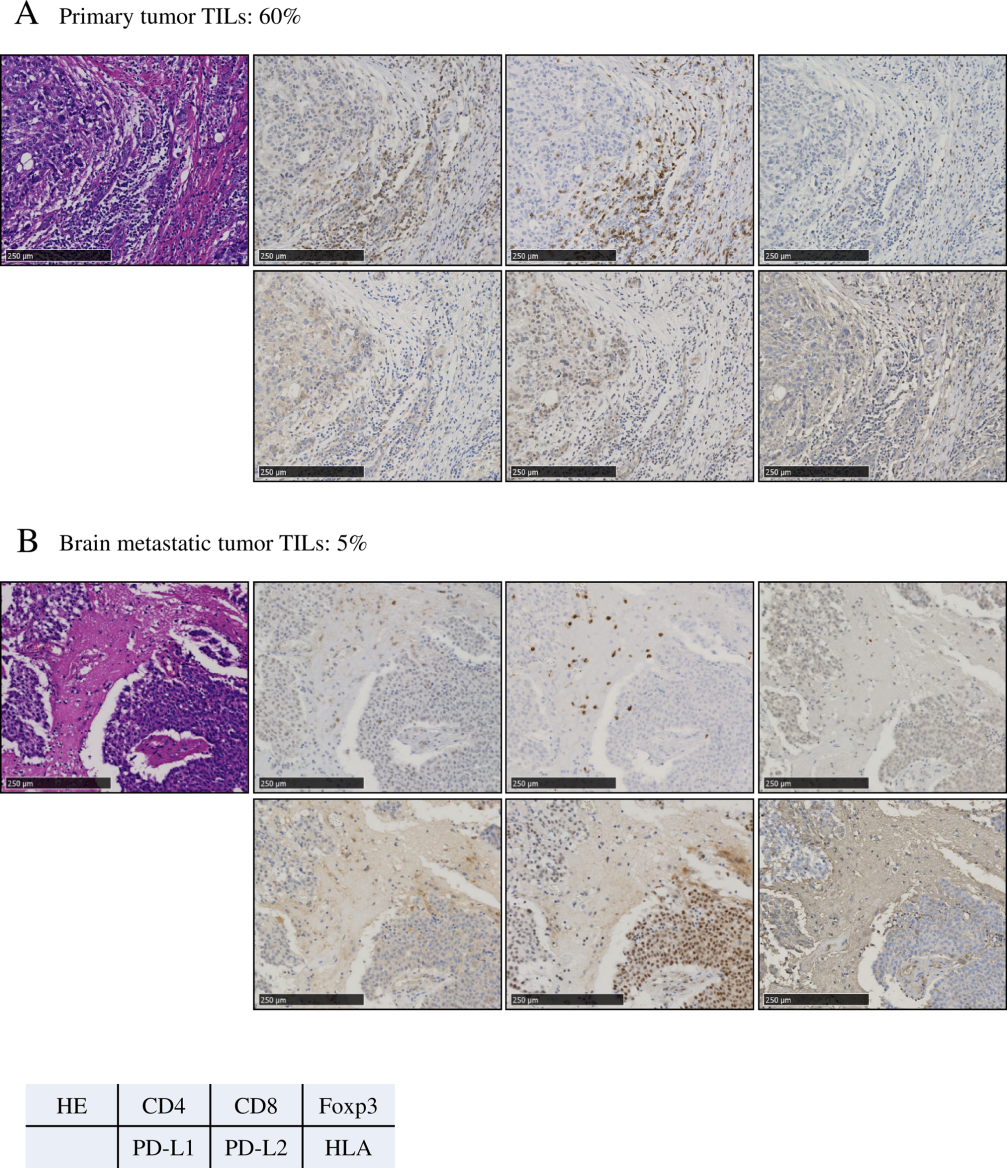
Representative photographs from a single patient with triple-negative primary breast cancer, showing the primary tumor **(A)** and brain metastatic tumor **(B)**. The expression of PD-L2 in the tumors converted from weak to strongly positive. Original magnification: ×400. (A) Primary tumor tumor-infiltrating lymphocytes (TILs): 60%. (B) Brain metastatic tumor TILs: 5%.

As for the pair-matched samples (*N* = 46), there were significantly more TILs in the primary breast tumors than in the brain metastases (paired *t*-test, *P* < 0.01) (Figure 1B). The numbers of CD4/CD8/Foxp3-positive cells were also significantly greater in the primary breast tumors than in the brain metastases (paired *t*-test, *P* < 0.05 [all categories]). There was a moderate positive correlation in the ratio of CD8/Foxp3-positive cells between primary and brain metastases tumors (Spearman’s correlation test, r = 0.406, *P* = 0.01). Representative images are shown in Figure 2.

Negative conversion of HLA expression in tumor cells was observed in brain metastases compared to the primary tumors (McNemer test, *P* = 0.06). In contrast, positive conversion of PD-L2 was observed in brain metastases compared to the primary tumors (McNemer test, *P* = 0.10). However, there was no clear difference in PD-L1 positivity between primary tumors and brain metastases (McNemer test, *P* = 0.58) (Supplementary Table 2).

**Figure 3:**
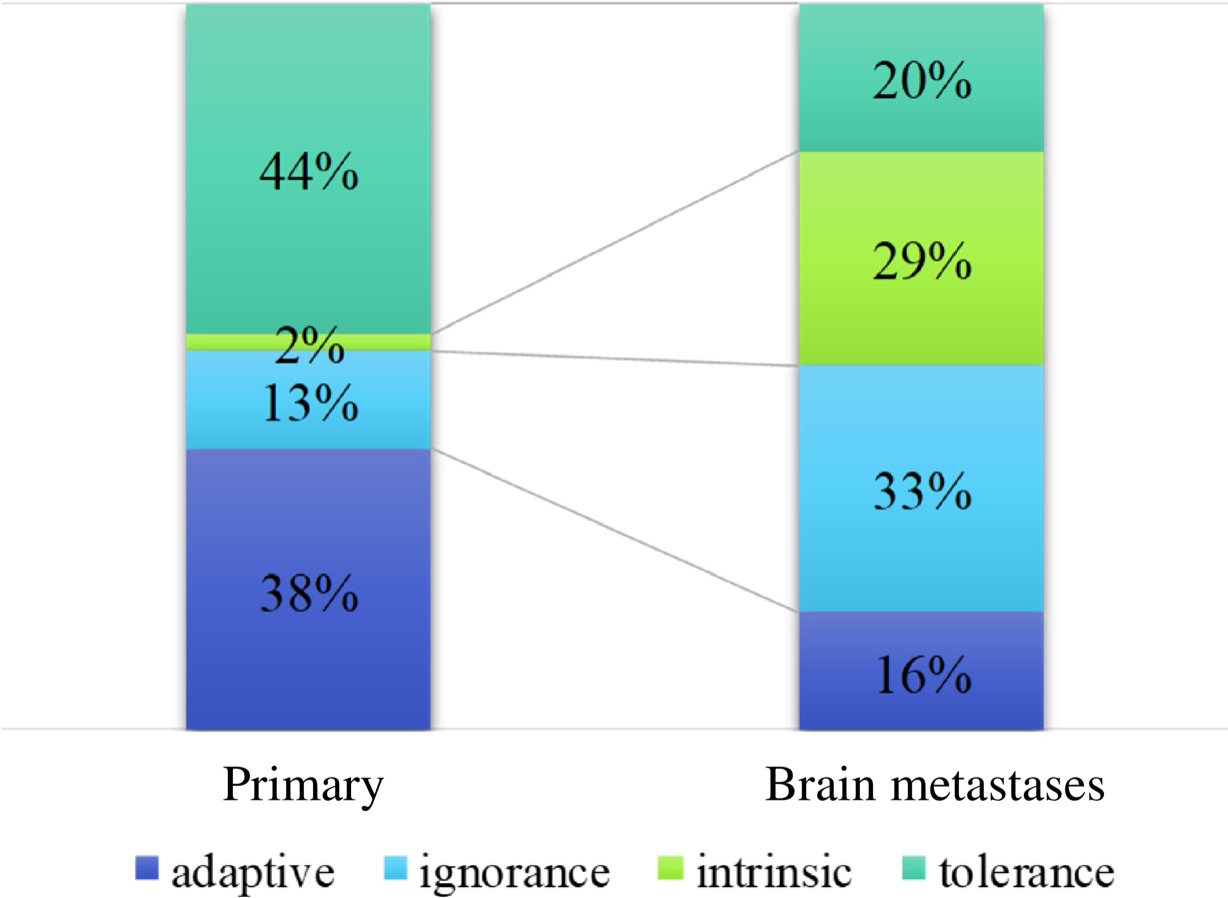
Four categories of immunologic features Type I (adaptive immune resistance, tumor-infiltrating lymphocyte [TILs] +/programmed death-ligand 1 [PD-L1] +), type II (immunological ignorance, TILs-/PD-L1-), type III (intrinsic induction, TILs-/PD-L1+), and type IV (tolerance, TILs+/PD-L1-).

As for brain metastasis classifications according to their microenvironments (described in the Patients and Methods section), 16% were type I, 17% were type II, 17% were type III, and 31% were type IV (*N* = 87). In pair-matched tumors, 42% of primary tumors were type I (adaptive) compared to 16% of brain metastases; moreover, 42% or primary tumors were type IV (tolerance) compared to 20% of brain metastases (Figure 3).

**Figure 4:**
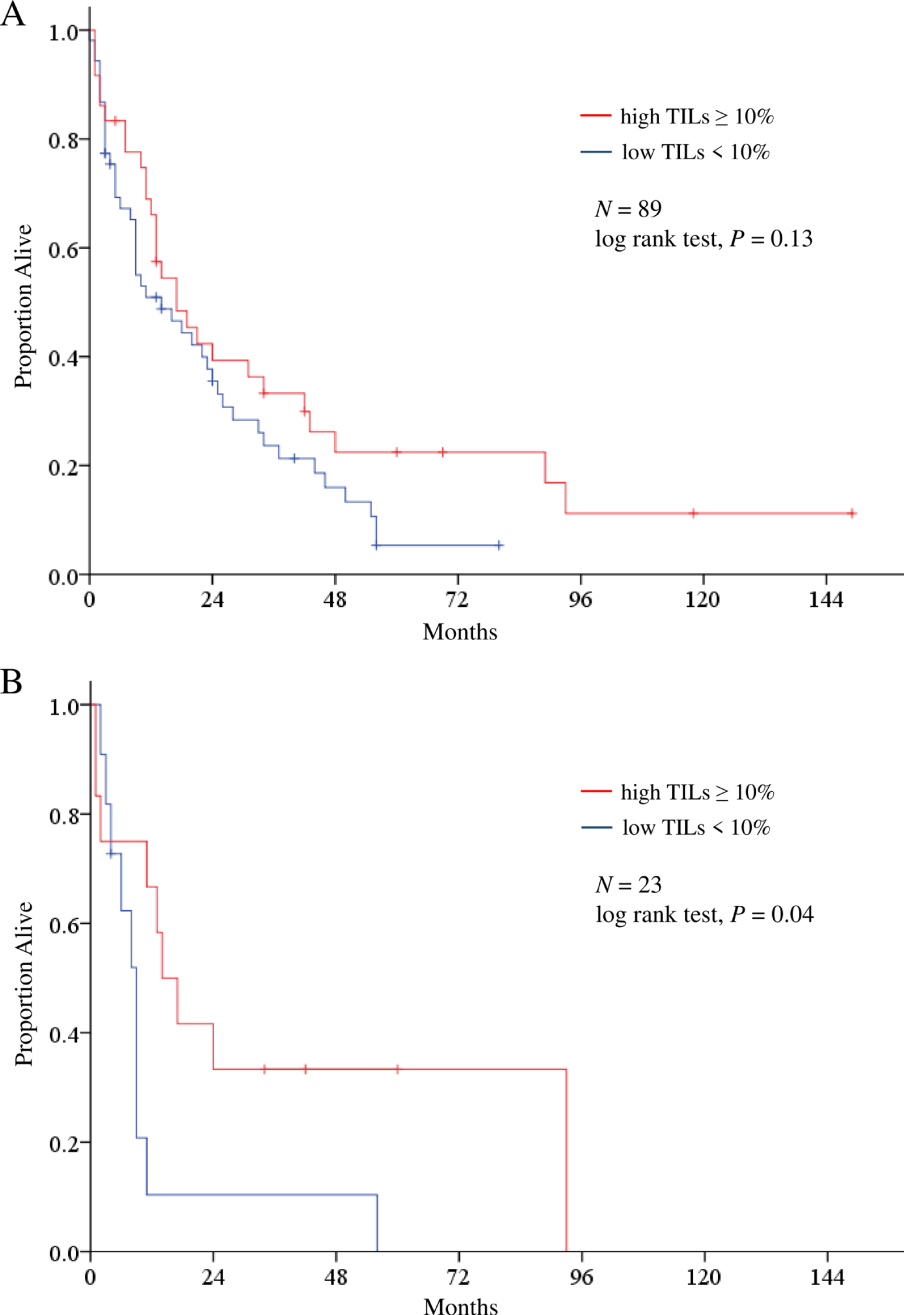
Kaplan-Meier estimates of overall survival following initial brain metastasis diagnosis according to the percentage of tumor-infiltrating lymphocytes (TILs) in brain metastases **(A)**, and subtype analysis for triple-negative breast cancer **(B)**.

We analyzed overall survival (OS) rates following the initial brain metastasis diagnosis according to the percentage of TILs in these brain metastases. Patients with low TILs had a shorter OS than those with high TILs (log-rank test, *P* = 0.131); the high/low cut-off point was the median percentage of TILs (Figure 4A). We next analyzed OS following the initial brain metastasis diagnosis according to the percentage of TILs in brain metastases by subtype (luminal HER2-negative, luminal HER2-positive, HER2-enriched, and TN); low TIL counts were associated with significantly shorter OS rates only in TN tumors (log-rank test, *P* = 0.04) (Figure 4B).

## DISCUSSION

In this study, we confirmed that brain metastases have fewer TILs compared to the primary breast tumors from which they arise. Our results are consistent with those of our previous study that compared the immune microenvironments of primary and metastatic breast tumors [15], and observed relatively fewer TILs in brain metastases compared to other metastatic sites. This may be attributed to the immune escape mechanisms of the tumor, as well as the discriminating immune environment of the brain.

Generally, the number of TILs in primary breast tumors is a prognostic factor in primary TN breast cancers [7]: HER2-positive and TN breast cancers demonstrate a higher rate of existing brain metastases (17 and 15%, respectively) compared to 9 and 11% for luminal A and B breast cancers, respectively [2]. We previously showed that TN breast cancer patients had poorer OS than did patients with other subtypes after they developed brain metastases [3]. We surmise that this occurs because TN breast cancer has a higher degree of malignancy than do other breast cancer subtypes. This has prompted a major drive towards the discovery of effective molecular therapeutic targets for TN breast cancer. Our data suggest that patients with low TIL counts in brain metastases have poorer prognoses than those with high counts. A clinical trial to test the effects of PD-L1 inhibitors in patients with brain metastases from epithelial-derived tumors is ongoing (NCT02669914).

In the present study, the expression levels of PD-L2 and HLA class I increased while those of HLA class I decreased in primary tumors vs. brain metastases. The transformation of the components of the brain metastasis milieu is consistent with the results of a previous study that showed that PD-L1 expression, TIL counts, or both were decreased in brain metastases compared to paired primary lung tumors [16]. The correlation between PD-L1 and PD-L2 expression on tumor cells differ among tumor types [17]. In a previous study, PD-L2 expression was independently associated with improved clinical outcomes in patients with head and neck squamous cell carcinoma [18]. Therefore, it is important to consider the spatial and temporal heterogeneity of the tumor immune microenvironment and the discordance between PD-L1 and PD-L2 expression in tumors when cancer patients are treated with PD-1 or PD-L1 inhibitors.

The proportions of the ‘adaptive immune resistance’ and ‘tolerance’ tumor types were lower in brain metastases than in the primary breast tumors. Sufficient T cell infiltration into tumors was critical for a response to PD-L1 blockade in a mouse model study [19]. Based on our results, the effects of PD-L1 inhibitor monotherapy for brain metastases may be limited in patients with breast cancer. The ‘intrinsic induction’ type, which comprised approximately 20% of brain metastases, may require combination therapy with an immune checkpoint inhibitor plus chemotherapy, radiotherapy, or different types of immune checkpoint inhibitors, which have the potential to induce lymphocyte infiltration. Conventional chemotherapy can stimulate the immune system against cancer in several ways, including directly activating CD4 positive, CD8 positive, or γδ T cells, leading to the production of chemokines and cytokines; inhibiting or depleting immunosuppressive myeloid-derived suppressor cells and regulatory T cells; and upregulating MHC class I expression on cancer cells [20]. Damaged cancer cells treated by radiotherapy release numerous chemokines and cytokines, and also upregulate MHC class I and PD-L1 expression on cancer cells [21, 22]. Some prospective clinical trials showed the safety and efficacy of immune checkpoint inhibitors combined with current standard chemotherapy [23]. Moreover, several retrospective series have shown that stereotactic radiation and cytotoxic T-lymphocyte–associated antigen 4 (CTLA-4) inhibitor or PD-1 inhibitor can be combined safely for melanoma patients with brain metastases [24, 25]. Moreover, CTLA-4 inhibitors frequently induce an increase in T cells within the tumor [26]. Therefore, identifying an optimal combination therapy involving immune checkpoint inhibition would be beneficial.

TILs in brain metastases have the potential to be affected by corticosteroids, which are generally prescribed to prevent brain edema. We attempted to compare the effect of corticosteroids between primary and brain metastases. However, there were few patients who had brain metastases at the diagnosis of breast cancer (*N* = 3); furthermore, the TIL expression percentages in these primary tumors were 70% in 1 case and 10% in 2 cases. Accordingly, further study concerning the effect of corticosteroids is required.

Our study has some limitations. First, it relied on retrospective data collected from multiple institutions. Second, regarding the pathological assessment, TIL evaluation was conducted by a single pathologist and tissues were processed in different laboratories before immunohistochemical analyses were performed, which may have led to variations in the results. Third, our study included patients who received different systemic treatments for brain metastases at multiple institutions, which may have affected the overall outcomes. Lastly, this study had a small sample size, as the number of surgeries performed for brain metastases has decreased due to advances in radiotherapy treatments. Hence, it will become increasingly difficult to collect tumor samples from brain metastases going forward.

In conclusion, we showed that brain metastases have decreased TIL counts compared to primary breast tumors. There was no significant difference in PD-L1 positivity between primary tumors and brain metastases.

## MATERIALS AND METHODS

### Patient samples

**Figure 5:**
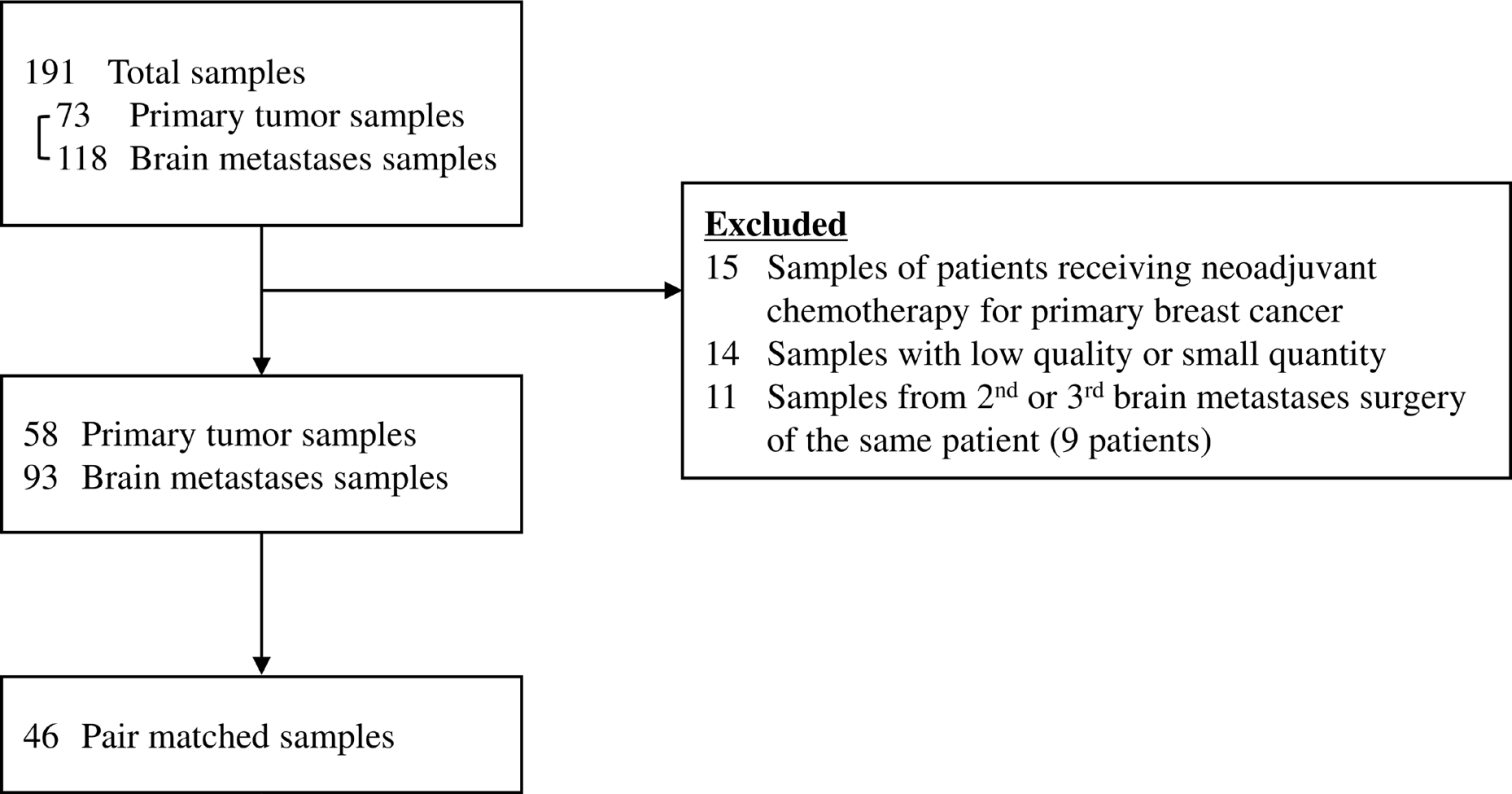
Flowchart showing the sample selection process

This investigation was based on a previous study performed by the Japan Clinical Oncology Group-Breast Cancer Study Group, which encompassed 34 clinical institutions in Japan. The eligibility criteria for the original study have been described previously [3]. A large dataset of 107 patients with breast cancer who were diagnosed with brain metastases and who underwent surgery between April 1, 2001 and December 31, 2012 was compiled from 8 institutions. Brain metastases were identified based on magnetic resonance imaging and/or computed tomography findings. A flowchart of the patient selection process is shown in Figure 5. Fourteen patients were found to have brain metastases when first diagnosed with breast cancer; the remaining patients were diagnosed with brain metastases subsequent to treatment for early or advanced breast cancer. We received 191 samples that included pair-matched samples of both the primary tumor and brain metastasis as well as brain metastasis samples only. Nine patients underwent 2 or more brain metastasis surgeries. We excluded 15 patients who received NAC because surgery specimens may contain a greater number of TILs after NAC administration than do specimens extracted before its initiation [9]. In the radiotherapy effect analysis, radiotherapy consisted of both whole brain irradiation and stereotactic irradiation. This study was approved by the institutional review board of each participating institute (Osaka National Hospital, Kindai University School of Medicine, Niigata Cancer Center Hospital, Tokai University School of Medicine, Shizuoka General Hospital, Hokkaido Cancer Center, National Hospital Organization, Tokyo Medical Center, Gunma Prefectural Cancer Center). The requirement for written informed consent was waived because of the retrospective nature of the study.

### Pathological assessment

We collected unstained slides from participating institutions and stained them at Tokai University. Samples stained with hematoxylin and eosin (H&E) were reviewed by a pathologist (N.K.) who was blinded to the clinicopathological characteristics of the patients, as recommended previously [27]. Representative H&E-stained slides that contained both the tumor and the stromal area were selected in each case and evaluated throughout the entire slide for TILs staining in stromal tissue adjacent to the primary breast tumor or in the brain parenchyma and stromal tissue adjacent to the brain metastases. The extents of staining were scored as 0–1% (considered 1%), > 1– < 10% (considered 5%), or in 10% increments between 10% and 100%. HER2-positive disease was referred to a score of 3+ on HER2 immunohistochemical analysis of the primary tumor or to amplification of the HER2 gene on fluorescence *in situ* hybridization. A patient was considered to have ER-positive and PR-positive disease if at least 1% of the tumor cells were positive for ER and PR on immunohistochemical analysis, respectively. Histological grading was assessed using the Nottingham grading system [28].

Immunohistochemistry was performed using primary antibodies to the following proteins: CD4, CD8, Foxp3, PD-L1, PD-L2, and HLA class I (Supplementary Table 1). To quantify the TILs in each antibody-stained slide, virtual slides were constructed using a Nano Zoomer 2.0 HT (Hamamatsu Photonics K.K., Hamamatsu, Japan) at 40× magnification. Three non-overlapping fields with average numbers of TILs on the H&E-stained slides were selected. Cells positive for each antibody were automatically counted using the ImageJ software (version 1.51a, NIH) [29, 30]. The numbers of cells positive for CD4, CD8, Foxp3, PD-L1, and PD-L2 in 3 fields were averaged. The expression of PD-L1, PD-L2, and HLA-class I-A, B, and C in the tumor cells was scored as 0 (negative), 1 (weak or focal [10% or less of the area was strongly positive]), or 2 (strong). We categorized scores of 0 and 1 as negative and scores of 2 as positive.

### Classification of tumors

Each specimen was categorized into 4 different types according to the presence of TILs and PD-L1 expression, as previously described [31]. This initial simple stratification of the tumors based on their immune reactivity sets a framework to identify which pathways should be targeted in order to elicit the best response for each tumor type. These are type I (adaptive immune resistance TILs-positive/ PD-L1-positive), type II (immunological ignorance, TILs-negative/PD-L1-negative), type III (intrinsic induction, TILs-negative/PD-L1-positive), and type IV (tolerance, TILs-positive/PD-L1-negative). We used the median percentage of TILs in brain metastases as the cut-off value (10%). Scores of 0 and 1 were considered negative, while 2 was considered positive for PD-L1 expression.

### Statistical methods

Paired *t*-tests for continuous variables and the McNemer test for categorical variables were used to determine differences in TIL counts and protein levels between primary tumors and brain metastases. Spearman’s test was used to determine the correlations between each category. OS was defined as the duration between the diagnosis of brain metastases and death of any cause or the last follow-up date and was calculated using the Kaplan–Meier method; survival curves were compared using the log-rank test. All statistical analyses were performed using IBM SPSS Statistics version 23 (Armonk, BY, USA); the differences were considered significant at *P* < 0.05.

## SUPPLEMENTARY MATERIALS TABLES



## Abbreviations

TILs: tumor-infiltrating lymphocytes
BBB: blood-brain barrier
NAC: neoadjuvant chemotherapy
HER2: human epidermal growth factor receptor-2
H&E: hematoxylin and eosin
TN: triple-negative,
ER: estrogen receptor
PR: progesterone receptor
PD-1: programmed death 1
PD-L1: programmed death-ligand 1
OS: overall survival
CTLA-4-: cytotoxic T-lymphocyte–associated antigen 4

## References

[1] Barnholtz-Sloan JS, Sloan AE, Davis FG, Vigneau FD, Lai P, Sawaya RE (2004). Incidence proportions of brain metastases in patients diagnosed (1973 to 2001) in the Metropolitan Detroit Cancer Surveillance System. J Clin Oncol.

[2] Rostami RS, Mittal P, Rostami F, Tavassoli B, Jabbari B (2016). Brain metastasis in breast cancer: a comprehensive literature review. J Neurooncol.

[3] Niikura N, Hayashi N, Masuda N, Takashima S, Nakamura R, Watanabe K, Kanbayashi C, Ishida M, Hozumi Y, Tsuneizumi M, Kondo N, Naito Y, Honda Y (2014). Treatment outcomes and prognostic factors for patients with brain metastases from breast cancer of each subtype: a multicenter retrospective analysis. Breast Cancer Res Treat.

[4] Galea I, Bechmann I, Perry VH (2007). What is immune privilege (not)?. Trends Immunol.

[5] Ningaraj NS (2006). Drug delivery to brain tumours: challenges and progress. Expert Opin Drug Deliv.

[6] Berghoff AS, Lassmann H, Preusser M, Hoftberger R (2013). Characterization of the inflammatory response to solid cancer metastases in the human brain. Clin Exp Metastasis.

[7] Loi S, Sirtaine N, Piette F, Salgado R, Viale G, Van Eenoo F, Rouas G, Francis P, Crown JP, Hitre E, de Azambuja E, Quinaux E, Di Leo A (2013). Prognostic and predictive value of tumor-infiltrating lymphocytes in a phase III randomized adjuvant breast cancer trial in node-positive breast cancer comparing the addition of docetaxel to doxorubicin with doxorubicin-based chemotherapy: BIG 02-98. J Clin Oncol.

[8] Denkert C, Loibl S, Noske A, Roller M, Muller BM, Komor M, Budczies J, Darb-Esfahani S, Kronenwett R, Hanusch C, von Torne C, Weichert W, Engels K (2010). Tumor-associated lymphocytes as an independent predictor of response to neoadjuvant chemotherapy in breast cancer. J Clin Oncol.

[9] Dieci MV, Criscitiello C, Goubar A, Viale G, Conte P, Guarneri V, Ficarra G, Mathieu MC, Delaloge S, Curigliano G, Andre F (2014). Prognostic value of tumor-infiltrating lymphocytes on residual disease after primary chemotherapy for triple-negative breast cancer: a retrospective multicenter study. Ann Oncol.

[10] Nanda R, Chow LQ, Dees EC, Berger R, Gupta S, Geva R, Pusztai L, Pathiraja K, Aktan G, Cheng JD, Karantza V, Buisseret L (2016). Pembrolizumab in Patients With Advanced Triple-Negative Breast Cancer: Phase Ib KEYNOTE-012 Study. J Clin Oncol.

[11] Khagi Y, Kurzrock R, Patel SP (2017). Next generation predictive biomarkers for immune checkpoint inhibition. Cancer Metastasis Rev.

[12] Goldberg SB, Gettinger SN, Mahajan A, Chiang AC, Herbst RS, Sznol M, Tsiouris AJ, Cohen J, Vortmeyer A, Jilaveanu L, Yu J, Hegde U, Speaker S (2016). Pembrolizumab for patients with melanoma or non-small-cell lung cancer and untreated brain metastases: early analysis of a non-randomised, open-label, phase 2 trial. Lancet Oncol.

[13] Duchnowska R, Peksa R, Radecka B, Mandat T, Trojanowski T, Jarosz B, Czartoryska-Arlukowicz B, Olszewski WP, Och W, Kalinka-Warzocha E, Kozlowski W, Kowalczyk A, Loi S (2016). Immune response in breast cancer brain metastases and their microenvironment: the role of the PD-1/PD-L axis. Breast Cancer Res.

[14] Harter PN, Bernatz S, Scholz A, Zeiner PS, Zinke J, Kiyose M, Blasel S, Beschorner R, Senft C, Bender B, Ronellenfitsch MW, Wikman H, Glatzel M (2015). Distribution and prognostic relevance of tumor-infiltrating lymphocytes (TILs) and PD-1/PD-L1 immune checkpoints in human brain metastases. Oncotarget.

[15] Ogiya R, Niikura N, Kumaki N, Bianchini G, Kitano S, Iwamoto T, Hayashi N, Yokoyama K, Oshitanai R, Terao M, Morioka T, Tsuda B, Okamura T (2016). Comparison of Tumour-infiltrating Lymphocytes between Primary and Metastatic Tumours in Breast Cancer Patients. Cancer Sci.

[16] Mansfield AS, Aubry MC, Moser JC, Harrington SM, Dronca RS, Park SS, Dong H (2016). Temporal and spatial discordance of programmed cell death-ligand 1 expression and lymphocyte tumor infiltration between paired primary lesions and brain metastases in lung cancer. Ann Oncol.

[17] Schmid P, Hegde PS, Zou W, Kowanetz M, Mariathasan S, Molinero L, Gadgeel SM, Powles T, Van Der Heijden MS, Fasso M, O’Hear C, Ballinger M, Fine GD (2016). Association of PD-L2 expression in human tumors with atezolizumab activity. J Clin Oncol.

[18] Yearley JH, Gibson C, Yu N, Moon C, Murphy E, Juco J, Lunceford J, Cheng J, Chow LQM, Seiwert TY, Handa M, Tomassini JE, McClanahan T (2017). PD-L2 Expression in Human Tumors: Relevance to Anti-PD-1 Therapy in Cancer. Clin Cancer Res.

[19] Tang H, Wang Y, Chlewicki LK, Zhang Y, Guo J, Liang W, Wang J, Wang X, Fu YX (2016). Facilitating T Cell Infiltration in Tumor Microenvironment Overcomes Resistance to PD-L1 Blockade. Cancer Cell.

[20] Galluzzi L, Senovilla L, Zitvogel L, Kroemer G (2012). The secret ally: immunostimulation by anticancer drugs. Nat Rev Drug Discov.

[21] Reits EA, Hodge JW, Herberts CA, Groothuis TA, Chakraborty M, Wansley EK, Camphausen K, Luiten RM, de Ru AH, Neijssen J, Griekspoor A, Mesman E, Verreck FA (2006). Radiation modulates the peptide repertoire, enhances MHC class I expression, and induces successful antitumor immunotherapy. J Exp Med.

[22] Dovedi SJ, Adlard AL, Lipowska-Bhalla G, McKenna C, Jones S, Cheadle EJ, Stratford IJ, Poon E, Morrow M, Stewart R, Jones H, Wilkinson RW, Honeychurch J (2014). Acquired resistance to fractionated radiotherapy can be overcome by concurrent PD-L1 blockade. Cancer Res.

[23] Antonia SJ, Gettinger SN, Goldman J, Brahmer J, Borghaei H, Chow LQ, Ready NE, Gerber DE, Juergens R, Shepherd F, Laurie SA, Young T, Geese WJ (2016). ORAL01.03: CheckMate 012: Safety and Efficacy of First-Line Nivolumab and Ipilimumab in Advanced NSCLC: Topic: Medical Oncology. J Thorac Oncol.

[24] Silk AW, Bassetti MF, West BT, Tsien CI, Lao CD (2013). Ipilimumab and radiation therapy for melanoma brain metastases. Cancer Med.

[25] Ahmed KA, Stallworth DG, Kim Y, Johnstone PA, Harrison LB, Caudell JJ, Yu HH, Etame AB, Weber JS, Gibney GT (2016). Clinical outcomes of melanoma brain metastases treated with stereotactic radiation and anti-PD-1 therapy. Ann Oncol.

[26] Huang RR, Jalil J, Economou JS, Chmielowski B, Koya RC, Mok S, Sazegar H, Seja E, Villanueva A, Gomez-Navarro J, Glaspy JA, Cochran AJ, Ribas A (2011). CTLA4 blockade induces frequent tumor infiltration by activated lymphocytes regardless of clinical responses in humans. Clin Cancer Res.

[27] Salgado R, Denkert C, Demaria S, Sirtaine N, Klauschen F, Pruneri G, Wienert S, Van den Eynden G, Baehner FL, Penault-Llorca F, Perez EA, Thompson EA, Symmans WF (2015). The evaluation of tumor-infiltrating lymphocytes (TILs) in breast cancer: recommendations by an International TILs Working Group 2014. Ann Oncol.

[28] Bloom HJ, Richardson WW (1957). Histological grading and prognosis in breast cancer; a study of 1409 cases of which 359 have been followed for 15 years. Br J Cancer.

[29] Schneider CA, Rasband WS, Eliceiri KW (2012). NIH Image to ImageJ: 25 years of image analysis. Nat Methods.

[30] Rasband WS http://imagej.nih.gov/ij/.

[31] Teng MW, Ngiow SF, Ribas A, Smyth MJ (2015). Classifying Cancers Based on T-cell Infiltration and PD-L1. Cancer Res.

