# Supplementary material for: Comparison of immune microenvironments between primary tumors and brain metastases in patients with breast cancer
Source: Oncotarget. 2017 Oct 27;8(61):103671–81. doi: 10.18632/oncotarget.22110 (PMC5732758; doi:10.18632/oncotarget.22110)
Supplement: Supplementary file 1 [file oncotarget-08-103671-s001.pdf]

Comparison of immune microenvironments between primary tumors and brain metastases in patients with breast cancer

SUPPLEMENTARY MATERIALS

Supplementary Table 1: Antibody and immunohistochemical assays

Marker	Type	Clone	Procedure	Manufacturer
CD4	Monoclonal	SP35	Manual	SPRING Bioscience (Pleasanton, CA)
CD8	Monoclonal	C8/144B	Manual	Nichirei (Tokyo, Japan)
Foxp3	Monoclonal	236A/E7	Manual	abcam (Cambridge, MA)
PD-L1	Polyclonal	ab58810	Autostainer	abcam
Pdcd-1L2 (PD-L2)	Monoclonal	XX19	Autostainer	SANTA CRUZ biotechnology (Dallas, TX)
HLA class I-A, B, C	Monoclonal	EMR8-5	Manual	HOKUDO (Sapporo, Japan)

PD-L1 and PD-L2 staining was performed using BOND-MAX (Leica) to improve detection sensitivity.

Supplementary Table 2: The comparison of expression on tumor cells

	Primary tumor (N)			Brain metastases (N)		
	**PD-L1**	**PD-L2**	**HLA**	**PD-L1**	**PD-L2**	**HLA**
negative	26	36	29	24	29	39
positive	18	8	15	20	15	6
